# Meat Microflora and the Quality of Meat Products

**DOI:** 10.3390/foods12091895

**Published:** 2023-05-05

**Authors:** Piotr Szymański, Dorota Zielińska, Anna Okoń, Anna Łepecka

**Affiliations:** 1 Department of Meat and Fat Technology, Prof. Waclaw Dabrowski Institute of Agriculture and Food Biotechnology—State Research Institute, 02-532 Warsaw, Poland; anna.okon@ibprs.pl (A.O.); anna.lepecka@ibprs.pl (A.Ł.); 2 Department of Food Gastronomy and Food Hygiene, Institute of Human Nutrition Sciences, Warsaw University of Life Sciences-SGGW, 02-776 Warsaw, Poland; dorota_zielinska@sggw.edu.pl

Meat and meat products are not only a source of nutrients for humans [1,2], but also an excellent substrate for the development of many microorganisms [3]. Fresh meat is always exposed to the action of many species of microorganisms, causing deterioration of its sensory quality and limiting its usefulness, both culinary and technological. The microbiological quality of meat is important both for consumers and from a safety point of view. Meat can be a habitat for saprophytic and pathogenic microorganisms that can deteriorate its quality or threaten the safety of consumers [1,4].

However, microorganisms present in meat products are not always a threat. Such microorganisms include lactic acid bacteria present in meat, which ferment sugars into lactic acid. This has a positive effect on the durability of the manufactured products. The presence and growth of lactic acid bacteria under controlled conditions have long been used in meat processing [5]. This enables the production of products with characteristic and desirable quality features, and at the same time with an extended shelf life. Running lactic acid fermentation processes in optimal conditions, however, often requires the use of highly selected microorganisms with precisely defined and stable characteristics. Such microorganisms are then deliberately introduced into meat in a certain amount during technological processes [5].

Due to the role played by starter cultures in meat products, they can be divided into the following groups: acidifying cultures, cultures supporting the curing process (denitrifying cultures) and stabilizing the curing color, cultures flavoring meat products and cultures stabilizing microbiological products (extending shelf life) [5,6].

A less durable raw material than the meat of animals is fish meat, which deteriorates faster and therefore should be frozen and stored at −20 °C. The cause of spoilage is most often psychrophilic microorganisms that develop at temperatures close to 0 °C. One way to improve the freshness and extend the shelf life of fish is multifunctional composite coatings. They are an interesting alternative to preserve the quality of fish fillets, but also to improve the quality of meat [7].

Appropriate use of selected strains of lactic acid bacteria may be useful in improving the microbiological quality of meat and meat products during storage. The purpose of this Special Issue was to compile original research and review papers covering various aspects of the impact of meat microflora on the quality characteristics and safety of meat and meat products.
